# P129: Experience of medical research institute (MRI) in applying WHO hand hygiene self assessment framework

**DOI:** 10.1186/2047-2994-2-S1-P129

**Published:** 2013-06-20

**Authors:** M Eldeeb, M Abo Ollo

**Affiliations:** 1 Clinical Chemistry, Alexandria, Egypt; 2 Medical research institute, Alexandria, Egypt

## Introduction

Many of infectious germs are transferred by hands while health-care providers or visitors are providing patient care. Using proper hand hygiene to keep hands clean is critical in order to reduce the risk of health care-associated infection.

## Objectives

Our aim was to identify the causes of noncompliance of hand hygiene in MRI health workers and co-workers in order to establish optimal hand hygiene behavior within a strong patient safety culture at MRI hospital.

## Methods

Situational analysis of hand hygiene practices at MRI hospital and departments were assessed using hand hygiene self assessment framework from 1^st^ to 15^th^ of April 2012. Results were sent to the WHO in order to put a corrective action plan to improve the overall hand hygiene performance of the MRI hospital.

At 5^th^ of May many activities were done in conjunction with the celebration of the International Day Of Hand Washing to increase the perception of health care workers about the importance of hand hygiene. Included. a)Internal audit for the medical team members on the performance of the right steps of hand washing. b)Awards were given to passing members.

## Results

Results of hand hygiene self assessment framework in conjugation with WHO helped us in putting corrective action plan to implement and sustain hand hygiene program at MRI hospital.

## Conclusion

The hand hygiene self assessment framework helped Infection Control Team to: a)Identify the weak points which hinder proper hand washing at MRI. b) Put corrective action plan & activities which help to improve the perception of hand hygiene system of health care workers.

## Disclosure of interest

M. Eldeeb Shareholder of participant in the programme, M. Abo Ollo Consultant for Head Of Infection Control Team

